# Editorial: Human Antibodies Against the Dietary Non-human Neu5Gc-Carrying Glycans in Normal and Pathologic States

**DOI:** 10.3389/fimmu.2020.01589

**Published:** 2020-07-23

**Authors:** Jean-Paul Soulillou, Vered Padler-Karavani

**Affiliations:** ^1^Centre de Recherche en Transplantation et Immunologie (CRTI), INSERM, Université de Nantes, Nantes, France; ^2^Institut de Transplantation Urologie Néphrologie (ITUN), CHU Nantes, Nantes, France; ^3^Department of Cell Research and Immunology, The George S. Wise Faculty of Life Sciences, Tel Aviv University, Tel Aviv, Israel

**Keywords:** cancer, xenotranplantation, sialic acid, *N*-glycolylneuraminic acid, inflammation, biotherapeutic, diet, carbohydrate

While most mammals commonly express the two forms of sialic acids, *N*-acetylneuraminic acid (Neu5Ac) and *N*-glycolylneuraminic acid (Neu5Gc), humans cannot synthesize Neu5Gc due to a loss-of-function mutation in the CMAH gene, which encodes the enzyme responsible for its synthesis. Consequently, Neu5Gc is immunogenic in humans, leading to generation of antibodies against various presentations of Neu5Gc-glycans. These antibodies appear in the first months of life and coincide with dietary intake of Neu5Gc (e.g., red meat and baby formulas containing cow's milk). Co-existence of Neu5Gc and anti-Neu5Gc antibodies in humans may have detrimental consequences, and in recent years, a considerable amount of fundamental information on this subject has been accumulated. Diet-derived Neu5Gc can be absorbed by human cells and can be found at very low levels on the surface of endothelial cells and of oncogenic epithelial cells. This unique situation results in the expression of a non-self carbohydrate in the context of self, coined as a “xeno-autoantigen.” Together with circulating anti-Neu5Gc “xeno-autoantibodies,” a peculiar “physiological” condition of chronic antibody exposure may lead to *in situ* chronic inflammation, termed xenosialitis, eventually contributing to various human diseases.

This Research Topic embeds a unique collection of papers summarizing current knowledge and remaining open questions regarding the possible consequences of this ongoing immune conflict between anti-Neu5Gc antibodies and Neu5Gc-glycans, during normal and pathological states. The first paper from the Varki group by Dhar et al. provides a historical perspective of this century-old enigma, rooted back in the early days when “serum sickness” was noticed. It also describes possible implications for diseases and therapy (Dhar et al.). Altman and Gagneux provide an evolutionary perspective for the loss of Neu5Gc expression in humans, and Angata puts that into context with host immune recognition of Neu5Gc-neoantigens by the siglecs immune receptors that respond to this evolutionary change. The reader is also referred to the recent historical review by the Center of Molecular Immunology (CIM, Cuba) related to human immunotherapy targeting the GM3(Neu5Gc) ganglioside (1). A major leap forward in research of Neu5Gc/anti-Neu5Gc was achieved owing to the newly developed tools for their investigation. In the paper by Kooner et al. from the Chen group, who pioneered these efforts, describe the chemistry behind the synthesis of designed natural and unnatural glycans with Neu5Gc and their reciprocal human Neu5Ac-containing glycans. Such glycans had been used to generate printed glycan microarrays focused on sialic acids that have dramatically advanced the field, as summarized by McQuillan et al. from the Cummings group. Beyond these tools, Breimer and Holgersson provide an overview of the structural complexity, diversity, and distribution of Neu5Gc-glycans in animal tissues, especially in the context of their immunogenicity and implications in clinical xenografting, further extended into the role of anti-Neu5Gc antibodies as an obstacle to xenotransplantation by Tector et al., while Perota and Galli describe the development of Neu5Gc-deficient large mammals (i.e., pigs and bovine) as a possible solution. It is commonly assumed that immune responses against Neu5Gc result from exposure to Neu5Gc-containing food items, however iatrogenic-induction also occur after treatment with Neu5Gc-containing animal-derived biotherpaeutics or bio-devices, yet their possible effects in autoimmune or other diseases remain poorly understood, as described by Yehuda and Padler-Karavani. In addition, Frei et al. discuss how rural lifestyle and increased anti-Neu5Gc antibodies levels could be protective against allergies. Despite this accumulated knowledge thus far, the mechanisms underlying the roles of anti-Neu5Gc antibodies particularly in human pathologies are largely unexplored, and most current investigations focus on several unresolved theoretical and fundamental questions directly related to a possible deleterious role of anti-Neu5Gc antibodies in humans. Thus, Soulillou et al. provide a critical perspective on current literature related to the suggested roles of anti-Neu5Gc antibodies in human pathologies. Altogether, this special issue is a major contribution to increase awareness of this very complex research related to the immunogenic Neu5Gc dietary carbohydrate in humans and its potential involvement with multiple human diseases.

## Author Contributions

All authors listed have made a substantial, direct and intellectual contribution to the work, and approved it for publication.

## Conflict of Interest

J-PS is the founder of Xenothera à French start-up dedicated to Neu5Gc knockout pig products, and collaborate with Avantea, a company with which they have produced Neu5Gc knockout cows. The remaining author declares that the research was conducted in the absence of any commercial or financial relationships that could be construed as a potential conflict of interest.
